# Graphene’s non-equilibrium fermions reveal Doppler-shifted magnetophonon resonances accompanied by Mach supersonic and Landau velocity effects

**DOI:** 10.1038/s41467-021-26663-4

**Published:** 2021-11-04

**Authors:** M. T. Greenaway, P. Kumaravadivel, J. Wengraf, L. A. Ponomarenko, A. I. Berdyugin, J. Li, J. H. Edgar, R. Krishna Kumar, A. K. Geim, L. Eaves

**Affiliations:** 1 Department of Physics, Loughborough University, Loughborough, LE11 3TU UK; 2 School of Physics and Astronomy, University of Nottingham, Nottingham, NG7 2RD UK; 3 School of Physics and Astronomy, University of Manchester, Manchester, M13 9PL UK; 4 National Graphene Institute, University of Manchester, Manchester, M13 9PL UK; 5 Department of Physics, University of Lancaster, Lancaster, LA1 4YW UK; 6 Tim Taylor Department of Chemical Engineering, Kansas State University, Manhattan, KS 66506 USA

**Keywords:** Electronic properties and devices, Electronic properties and materials

## Abstract

Oscillatory magnetoresistance measurements on graphene have revealed a wealth of novel physics. These phenomena are typically studied at low currents. At high currents, electrons are driven far from equilibrium with the atomic lattice vibrations so that their kinetic energy can exceed the thermal energy of the phonons. Here, we report three non-equilibrium phenomena in monolayer graphene at high currents: (i) a “Doppler-like” shift and splitting of the frequencies of the transverse acoustic (TA) phonons emitted when the electrons undergo *inter*-Landau level (LL) transitions; (ii) an *intra*-LL Mach effect with the emission of TA phonons when the electrons approach supersonic speed, and (iii) the onset of elastic inter-LL transitions at a critical carrier drift velocity, analogous to the superfluid Landau velocity. All three quantum phenomena can be unified in a single resonance equation. They offer avenues for research on out-of-equilibrium phenomena in other two-dimensional fermion systems.

## Abstract (web summary)

Magneto-oscillations have revealed many interesting phenomena in graphene and quantum Hall systems, but they are typically measured at low currents and in equilibrium. Here, the authors report several non-equilibrium quantum effects observed in magneto-oscillations in graphene at high currents.

## Introduction

Non-equilibrium phenomena in conventional semiconductors are of fundamental interest and have been exploited for technology, for example, high performance GaAs Gunn oscillators^1^ and quantum cascade lasers^2^. They are anticipated to be particularly prominent in graphene due to its weak electron–phonon coupling.

The effect of high currents and strong electric fields, **F**, on the magnetoresistance of Landau-quantised electrons in semiconductors has been investigated intensively. In bulk n-type GaAs, fields *F* ~ 10^6^ Vm^−1^ induce large shifts and splittings of the magnetophonon resonance (MPR) peaks due to scattering by 36 meV longitudinal optical phonons, along with quasi-elastic inter-Landau level (LL) transitions^3–5^. In contrast, the field-induced shifts and splittings in high-mobility GaAs quantum well (QW) heterostructures arise from inter-LL scattering by acoustic phonons of lower energy (a few meV)^6–9^. A competing process due to elastic inter-LL interactions at high currents also give rise to Hall field-induced oscillations, which have been investigated in QWs composed of GaAs^10–12^, Ge/SiGe^13^, and MgZnO/ZnO^14^. It is interesting to note that in a GaAs QW, inter-LL processes can also manifest themselves as a breakdown of the integer quantum Hall effect^15–19^.

For graphene, resonant magnetoresistance measurements under “ohmic” low current conditions have been used to study the quantum Hall and flux quantisation effects^20–25^. Non-equilibrium phenomena at higher currents are anticipated to be particularly prominent due to graphene’s relatively weak electron–phonon coupling. Furthermore, the relativistic fermions in graphene have an order of magnitude higher group velocity than electrons in conventional semiconductors. Similarly, graphene’s acoustic and optical phonon modes have much higher speeds and larger energies. Graphene’s ability to support large current densities of over ~100 Am^−1^ (or equivalently ~10^11^ Am^−2^) enables us to explore resonant hot carrier phonon emission together with the formation and dissipation of magneto-excitons^19,26–29^. We show that all of the observed phenomena can be unified in terms of a single generic resonant scattering equation.

## Results and discussion

### MPR splitting at high currents

**Fig. 1:**
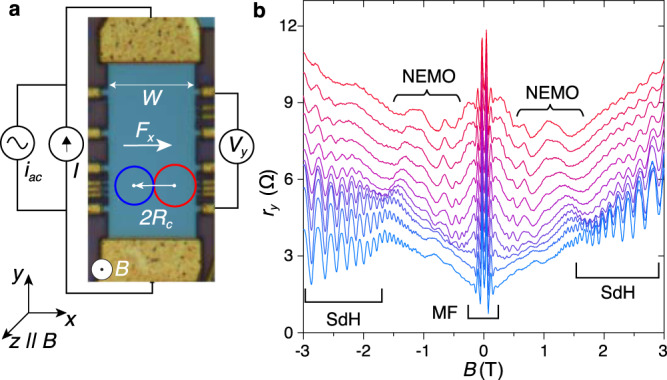
Current dependence of magnetoresistance oscillations in monolayer graphene Hall bars. **a** Optical micrograph image of the graphene Hall bar (*W* = 15 μm) and a schematic diagram of the measurement configuration. **b** Plots of differential resistance *r*_*y*_ = d*V*_*y*_/d*I* at *T* = 5 K as a function of *B* for DC currents, *I*, between 0 (blue) and 140 μA (red) in 14 μA intervals, curves are offset by 0.7 Ω for clarity. Curly brackets indicate the emergence of additional non-equilibrium magneto-oscillations (NEMOs) examined in detail in Figs. 2 and 3. Square brackets labelled SdH indicate Shubnikov–de Haas oscillations and square brackets labelled MF indicate the magnetic focussing peaks.

We search for non-equilibrium magneto-oscillations (NEMOs) using large area Hall bars of high-purity exfoliated monolayer graphene encapsulated between layers of exfoliated hexagonal boron nitride and mounted on a silicon oxide–silicon gate electrode; for further information regarding fabrication, see ref. ^30^. An image of the device is shown in Fig. 1a. Recent work has demonstrated that these devices, with dimensions, ∼10 μm,  in excess of the phonon-limited mean free path, are required to reveal MPRs in graphene^30,31^. Those measurements were made at temperatures up to 200 K under ohmic conditions at small direct currents (DCs), so the carriers remained in equilibrium with the lattice phonons.

Figure 1a shows our circuit arrangement. In parallel with a small low frequency modulation current, *i* = 2 μA, a DC current, *I*, up to *I* = 1 mA is passed through a multi-terminal Hall bar of width *W* = 15 μm. The differential magnetoresistance *r*_*y*_ = d*V*_*y*_/d*I* is measured using a lock-in amplifier as a function of *I* and applied magnetic field, *B*.

**Fig. 2:**
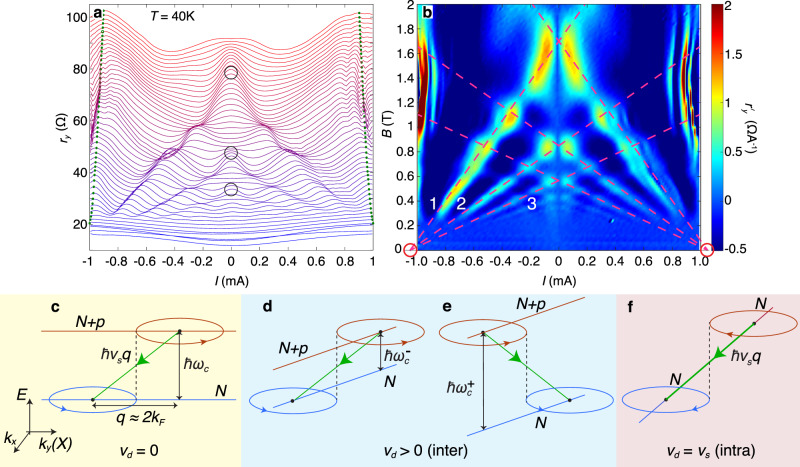
Non-equilibrium magnetoresistance oscillations at *T* = 40 K: magnetophonon resonance splitting and the Mach effect. **a** Plot of differential resistance *r*_*y*_(*I*) for *B* between 0 and 2 T in 0.04 T intervals and **b** the corresponding colour map of ${r}_{y}^{\prime}(I,B)=-{{{{{{{\rm{sgn}}}}}}}}(I){{{{{\rm{d}}}}}}{r}_{y}/{{{{{\rm{d}}}}}}I$, measured at *T* = 40 K and *n* = 3.16 × 10^12^ cm^−2^. In **a**, the curves are offset by 1.5 Ω and the open circles indicate the magnetophonon resonance peaks *p* = 1, 2, 3 under ohmic conditions when *I* = 0. The green markers show the position of the peak in the resonance at *v*_d_ ≈ *v*_s_ given by Supplementary Eq. (1). In **b**, the dashed magenta lines are a fit given by Eq. (7) when *v*_*s*_ = *v*_TA_ and *p* = 1, 2, 3 (labelled). The red circles at the left and right hand corners of the colour map highlight the point of convergence of the magenta lines when *B* = 0. **c**–**f** show energy–momentum diagrams in applied *B* and *F*_*x*_ to demonstrate the semiclassical conditions for strong scattering between initial (red, *N* + *p*) and final (blue, *N*) Landau states when: **c**, *v*_*d*_ ≈ 0, corresponding to ohmic MPR; **d**, **e** *v*_d_ < *v*_s_, corresponding to Hall field-induced splitting of the MPR features when $B={B}_{p}^{-}$ (**d**) and $B={B}_{p}^{+}$ (**e**). **f** *v*_d_ = *v*_s_, corresponds to intra-LL acoustic phonon scattering. In **c**–**f**, the red and blue circles show the semiclassical trajectories of the carriers in *k-*space, and the red and blue lines show the energies of the initial and final Landau states and their dependence on *k*_*y*_(*X*) in the finite Hall field. The dashed vertical line shows the points of intersection of the semiclassical figure-of-8 orbits when *q* = 2*k*_F_. The arrowed green lines show the phonon-assisted transitions between LLs, **c**–**e**, and within a single LL, **f**.

**Fig. 3:**
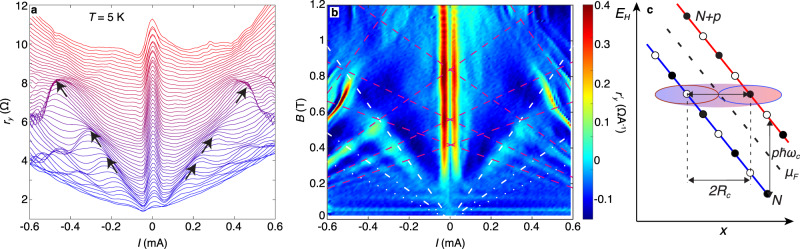
Non-equilibrium magnetoresistance oscillations at *T* = 5 K: elastic inter-LL transitions. **a** Plot of *r*_*y*_(*I*) for values of *B* between 0 to 1.2 T in 0.04 T intervals when *T* = 5 K and *n* = 3.16 × 10^12^ cm^−2^. Curves are offset by 1.5 Ω for clarity. Black arrows highlight position of peaks in *r*_*y*_. **b** Colourmap of ${r}_{y}^{\prime}(I,B)$ when *T* = 5 K. Dashed white lines show the linear dependence of the *B*_*p*_ on *I*, see Eq. (10), for the phononless resonances *p* = 1, 2, 3. In **b**, the magenta lines are a fit given by Eq. (7) for the Hall-field-dependent MPR peak with *p* = 1, 2, 3, 4. **c** shows how elastic inter-LL transitions can create a non-equilibrium distribution of carriers involving interacting electron–hole pairs, “magneto-excitons”.

Figure 1b shows the dependence of *r*_*y*_ on *B* and *I* at a bath temperature of *T* = 5 K and a gate voltage *V*_g_ = −60 V, which generates a carrier sheet density (holes) of *n* = 3.16 × 10^12^ cm^−2^. The large amplitude features close to *B* = 0, which persist up to currents of at least ~250 μA and over a wide range of *n* (see Supplementary Fig. 1) are due to well-established magnetic focussing (MF) of the quasi-ballistic carriers^30,32^. They indicate that our carefully exfoliated devices are largely free from disordered localised states. At low currents, strong Shubnikov–de Haas (SdH) oscillations appear at *B* ≳ 1 T. They damp out as *I* is increased and the carrier distribution becomes non-thermal and broadened around the Fermi level, *μ*_F_. In their place, a new set of resonant magneto-oscillations appear (NEMO, see Fig. 1b) whose behaviour and origin we explore in Figs. 2 and 3.

The effect on the magnetoresistance oscillations of the Hall field, *F*_*x*_, induced by the DC current is illustrated in Fig. 2a, b, which plot a set of *r*_*y*_(*I*) curves versus *B*. At *T* = 40 K and *I* = 0, the peaks in *r*_*y*_ (circled), which are periodic in 1/*B*, correspond to ohmic MPR oscillations due to transverse acoustic (TA) phonon scattering transitions, as reported recently^30,31^ and illustrated schematically in Fig. 2c. With increasing *I* and *F*_*x*_, the magnetic field positions of the MPRs undergo large shifts and splittings. These features are highlighted by magenta dashed lines in Fig. 2b, which plots the second derivative of *V*_*y*_ with respect to *I*, ${r}_{y}^{\prime}=-{{{{{{{\rm{sgn}}}}}}}}(I){{{{{\rm{d}}}}}}{r}_{y}/{{{{{\rm{d}}}}}}I$.

The splittings and shifts arise from the effect of *F*_*x*_, which tilts the energies of the quantised Landau states, see Fig. 2d–f. Graphene’s high carrier mobility, *μ* ~ 50 m^2^ V^−1^ s^−1^, provides a large value of *μB* ≫ 1, thus ensuring Landau quantisation of well-defined cyclotron orbits at fields as low as *B* ~ 0.3 T. When *F*_*x*_ = 0, a carrier in a LL with index *N* has an energy ${E}_{N}=\hbar \sqrt{2N}{v}_{{{{{{\rm{F}}}}}}}/{l}_{B}$, where ${l}_{B}=\sqrt{\hbar /eB}$ is the magnetic length and *v*_F_ is the Fermi velocity in graphene. When *F*_*x*_ ≠ 0, the energy of a carrier depends on the location of its real-space orbit centre *X* across the width of the Hall bar and thus on its wave-vector component, *k*_*y*_, along the *y* axis: $X({k}_{y})=-{k}_{y}{l}_{B}^{2}$, in the Landau gauge. Therefore, under the condition *v*_d_ ≪ *v*_F_, which is satisfied in our experiment, the energy of a carrier in LL index *N* is given by^33,34^:

1 $${E}_{N}({k}_{y})={E}_{N}-e{F}_{x}X({k}_{y}).$$

A carrier can scatter inelastically between LL states with indices *N* and (*N* + *p*) and wavevectors *k*_*y*_ and ${k}_{y}^{\prime}$ by emitting or absorbing an acoustic phonon of speed *v*_s_ and wavevector *q* governed by the relation

2 $${E}_{N+p}({k}_{y}^{\prime})-{E}_{N}({k}_{y})=\hbar {v}_{{{{{{\rm{s}}}}}}}q.$$

To a good approximation, the energy gap between LLs is given by the correspondence principle when *N* is large and *p* is small:

3 $${E}_{N+p}-{E}_{N}=p\hbar {\omega }_{{{{{{\rm{c}}}}}}}.$$

Here, *p* = 1, 2, 3. . . , *ω*_c_ = *eB*/*m*_c_ is the cyclotron frequency and *m*_c_ = $\hbar$*k*_F_/*v*_F_ is the wavevector-dependent cyclotron mass for Landau states close to the Fermi energy, where *N* ≈ 30 at *B* = 1 T.

Scattering between Landau states is strongest when the spatial overlap of their wavefunctions and the transfer of momentum is at a maximum. Semiclassically, this occurs when the two cyclotron orbits of the initial and final states just touch to form a “figure-of-8” scattering configuration, see Figs. 1a and 2c–f^31,35^. The separation between the two orbit centres is given by 2*R*_c_, where ${R}_{{{{{{\rm{c}}}}}}}={l}_{B}^{2}{k}_{{{{{{\rm{F}}}}}}}$ is the classical cyclotron radius at the Fermi energy and ${k}_{{{{{{\rm{F}}}}}}}=\sqrt{\pi n}$ is the Fermi wavevector. For such a transition, the carrier’s momentum is changed by 2$\hbar$*k*_F_ along the *x*-axis.

### Doppler-shifted frequencies of emitted phonons

Referring to Fig. 2d, e, and using Eqs. (1)–(3) with the phonon wavevector, *q* = 2*k*_F_, we derive a generic condition for current-dependent magnetophonon scattering between LLs:

4 $$p{\omega }_{{{{{{\rm{c}}}}}}}=2{k}_{{{{{{\rm{F}}}}}}}({v}_{{{{{{\rm{s}}}}}}}\pm {v}_{{{{{{\rm{d}}}}}}}),$$

where *v*_d_ = *F*_*x*_/*B* is the carrier drift velocity along the Hall bar. To a good approximation

5 $${v}_{{{{{{\rm{d}}}}}}}=\frac{I}{enW},$$

since *μB* ≫ 1. In the limit when *I* = *v*_d_ = *F*_*x*_ = 0, we obtain the ohmic MPR condition^30,31^, *pω*_c_ = 2*v*_s_*k*_F_ or equivalently

6 $${B}_{p}({v}_{{{{{{\rm{d}}}}}}}=0)={B}_{p}^{0}=\frac{hn{v}_{{{{{{\rm{s}}}}}}}}{pe{v}_{{{{{{\rm{F}}}}}}}}.$$

The measured values of ${B}_{p}^{0}$ and their dependence on *n* give the value of *v*_s_^30^ and confirm that the resonances are due to TA phonon scattering with *v*_s_ = *v*_TA_ = 1.36 × 10^4^ m s^−1^. Equation (4) can be rewritten to give the magnetic field position of each resonance in terms of *I*:

7 $${B}_{p}(I)={B}_{p}^{0}\left(1\pm \frac{I}{{v}_{s}enW}\right).$$

Thus, for a given *I* and *p* there are two resonant conditions with magnetic field positions *B*_*p*_(*I*)^+^ and *B*_*p*_(*I*)^−^. They correspond to scattering of carriers either in the positive or negative *x-*direction for which the electrostatic potential energy, *eF*_*x*_*x*, increases or decreases. The shifts of the phonon frequencies, *ω*_s_, are found by rearranging Eq. (4) to give *ω*_s_ = 2*k*_F_*v*_s_ = *pω*_c_ ± 2*k*_F_*v*_d_, see also Fig. 2d, e. These shifts can be thought of as a modified form of the Doppler effect, in which phonon quanta of the TA sound waves are preferentially emitted perpendicular to the Hall field.

As shown in Fig. 2b, the dashed magenta lines corresponding to relation (7) provide a good fit to the loci and splitting of the measured MPRs in ${r}_{y}^{\prime}$. Over this range of *I* and *B* with *n* = 3.16 × 10^12^ cm^−2^, the inelastic resonant transitions between partially filled LLs occur around *μ*_F_ = 210 meV, with large LL indices (*N* ~ 33 at *B* = 1 T) and with approximately equal LL spacing $\hbar$*ω*_c_ = *ev*_F_*B*/*k*_F_ ~ 3 meV. A notable feature in Fig. 2b is that the peaks in ${r}_{y}^{\prime}$ are strongest at the crossing points of the magenta lines when ${B}_{p^{\prime} }^{+}={B}_{p}^{-}$ so that two different energy relaxation routes are available.

### Supersonic electrons and a Mach effect

We consider next the strong and broad peak in *r*_*y*_ in Fig. 2a centred at *I* ≈ ± 0.9 mA over a wide range of *B* from ~0.5 to 2 T. The position of the peak is marked by the near-vertical set of green dots in Fig. 2a, see Supplementary Note 1. Beyond this value of ∣*I*∣, *v*_d_ ≥ 1.19 × 10^4^ m s^−1^ and approaches the measured value of *v*_TA_ = 1.36 × 10^4^ m s^−1^ in this device^30,31^. This leads to the onset of strong *intra*-LL scattering of fast-drifting carriers and the emission of TA phonons, including those with large momentum and wavevector *q* ≈ 2*k*_F_ (see Fig. 2f), which make a major contribution to the magnetoresistance. For such an intra-LL transition, the energy conservation condition for phonon emission is independent of the energy separation, $\hbar$*ω*_c_, of the LLs. This results in the broad, large amplitude peak in *r*_*y*_ at *I* ≈ ±0.9 mA with increasing *B* from 0.5 to 2 T. This corresponds to the generic resonance condition, Eq. (4) with *p* = 0 so that

8 $$\frac{I}{enW}={v}_{{{{{{\rm{d}}}}}}}={v}_{{{{{{\rm{s}}}}}}}$$

is independent of *B*. This strong peak over such a wide range of *B* in monolayer graphene represents a two-dimensional (2D) analogy with Mach’s “supersonic boom” effect^7,8,16,36^. In this regime, the electric field effectively acts as an energy pump for amplified phonon emission by the hot carriers^37–39^. Note that as ∣*v*_d_∣ → *v*_s_ then *B*_*p*_(*I*)^−^ → 0, see Eq. (7). This is confirmed by the convergence of the shifted MPR peaks in ${r}_{y}^{\prime}$ at the points *I* = ±1.05 mA and *B* = 0, red circles in Fig. 2b. The magenta lines in Fig. 2b highlight this convergence. The linewidth, ${{\Gamma }}(B)\propto \sqrt{B}$, of the peak has a weak *B*-dependence similar to previous experiments and theoretical work on graphene^40^, see Supplementary Note 1.

### Elastic inter-LL transitions

At low temperatures (*T* = 5 K), we observe a third type of non-equilibrium resonant phenomenon, which is revealed by the well-defined resonant peaks in *r*_*y*_ highlighted by black arrows in Fig. 3a. The dashed white lines in the ${r}_{y}^{\prime}$ plot in Fig. 3b indicate the strong peaks, which are periodic in 1/*B* and shift linearly with a slope d*B*/d*I* ≈ ±1.6 T mA^−1^. Two weaker peaks (dot-dashed lines) shift at rates of 0.8 T mA^−1^ and ≈0.5 T mA^−1^. These peaks have a quite different character from the shifts of the MPR peaks observed at 40 K in Fig. 2. We now demonstrate that this third type of resonance arises from energy-conserving transitions between LLs, analogous to Zener tunnelling^41^, as shown schematically in Fig. 3c. They correspond to the semiclassical “figure-of-8” condition when *p*$\hbar$*ω*_c_ = 2*k*_F_*v*_d_ = 2*R*_c_*eF*_*x*_ (i.e. generic equation Eq. (4) with *v*_s_ = 0) from which we obtain a critical carrier drift velocity

9 $${v}_{{{{{{\rm{c}}}}}}}=p\frac{{\omega }_{{{{{{\rm{c}}}}}}}}{2{k}_{{{{{{\rm{F}}}}}}}}.$$

The resonances occur at magnetic field *B*_*p*_, which shifts linearly with current:

10 $${B}_{p}=\frac{hn{v}_{{{{{{\rm{d}}}}}}}}{pe{v}_{{{{{{\rm{F}}}}}}}}=\frac{h}{p{e}^{2}{v}_{{{{{{\rm{F}}}}}}}W}| I| .$$

The white lines in Fig. 3b given by Eq. (10) with *p* = 1, 2 and 3 and *v*_F_ = 1.05 × 10^6^ m s^−1^ provide a good fit to the measured linear *B*(*I*) dependence of the resonant peaks in ${r}_{y}^{\prime}$. Note that Eq. (10) is independent of *n* (see also Supplementary Note 2) and involves only one fitting parameter, *v*_F_; therefore, our measurements provide an independent method to determine the Fermi velocity in graphene. This measurement of *v*_F_ is arguably more direct than that obtained by modelling the temperature-dependent damping of SdH oscillations^20^. We suggest that NEMO measurements combined with Eq. (9) could be useful to determine the cyclotron effective mass of other 2D materials.

### Magneto-excitons

These elastic phononless inter-LL transitions require a mechanism, such as defect-induced scattering^3,34,42^, that enables transitions between the orthogonal initial and final states. In our encapsulated graphene Hall bars, the high carrier mobility, long mean free path^30^ and the presence of MF peaks in the magnetoresistance, labelled MF in Fig. 1b, suggest that defect-induced carrier scattering is relatively weak. Therefore, we propose a particular type of electron–electron interaction, namely, the formation of magneto-excitons^19,26–29^, as a likely contributor to the strong resonant processes described by Eq. (10). Magneto-excitons are quasi-particles that comprise an interacting electron and hole pair in energetically adjacent LLs. They are connected with the poles in the polarisability function, Π(*ω*, *q*), at *q* = 0 and *ω* = *pω*_c_(*p* = 1, 2. . . )^26^. In the absence of an electric field, the magneto-exciton dispersion is gapped so that ${\omega }_{{{{{{\rm{ME}}}}}}}^{p}(q,{v}_{{{{{{\rm{d}}}}}}}=0)\, > \, p{\omega }_{{{{{{\rm{c}}}}}}}$, and there is a low probability for exciton formation. However, in a strong Hall field, the magneto-exciton dispersion is modified by the term *v*_d_*q*:

11 $${\omega }_{ME}^{p}(q,{v}_{d})={\omega }_{ME}^{p}(q,{v}_{d}=0)-{v}_{d}q.$$

When $q \, > \, {\omega }_{{{{{{\rm{ME}}}}}}}^{p}(q,{v}_{{{{{{\rm{d}}}}}}}=0)/{v}_{{{{{{\rm{d}}}}}}}\approx {\omega }_{{{{{{\rm{c}}}}}}}/{v}_{{{{{{\rm{d}}}}}}}$, ${\omega }_{{{{{{\rm{ME}}}}}}}^{p}(q,{v}_{{{{{{\rm{d}}}}}}})$ is negative, and the magneto-exciton gap closes^29^.

A magneto-exciton with a particular *q* can only form when its spectral density is non-zero which, within a single particle picture, corresponds to a strong overlap of the electron and hole wavefunctions of the *N* and (*N* + *p*) LL states^29^. The onset of this condition corresponds to the “figure-of-8” configuration shown in Fig. 3c in which magneto-excitons form with a length scale ≈ 2*R*_*N*_ and wavevector *q* ≈ 2*k*_F_. Thus, when *v*_d_ ≳ *ω*_c_/2*k*_F_ = *v*_c_ spontaneous formation and proliferation of magneto-excitons can occur. Strong evidence for such a process has been detected as shot noise in quantum Hall effect breakdown in bilayer graphene^29^. A magneto-exciton can decay by dissociation in a Hall field or by scattering of the component charges into nearby LL states with lower energy, accompanied by the emission of phonons, thus generating the dissipative voltage *V*_*y*_, which gives rise to the resonant magnetoresistance peaks in Fig. 3a, b. The enhancement of the resonances in *r*_*y*_ and ${r}_{y}^{\prime}$, revealed in Fig. 3b at the intersections of the conditions for Hall-field-shifted MPR (magenta lines) and magneto-exciton formation (dashed white lines), can then be explained by the combined effect of magneto-exciton formation and decay by phonon emission.

Finally, we note that the formation of magneto-excitons when *v*_d_ ≥ *v*_c_ = *pω*_c_/2*k*_F_, see Eq. (9), is somewhat analogous to the formation of quasi-particles and dissipation in superfluids^27,43^ and that the expression for our critical velocity, *v*_c_, resembles the critical Landau velocity, given by the energy–momentum ratio of a superfluid quasi-particle. For Landau-quantised carriers in graphene, the circulation, *κ*, around a cyclotron orbit is given by:

12 $${\kappa }_{N}=\oint {{{{{{{\bf{v}}}}}}}}.{{{{{\rm{d}}}}}}{{{{{{{\bf{l}}}}}}}}=2\pi {R}_{{{{{{\rm{c}}}}}}}{v}_{{{{{{\rm{F}}}}}}}=\frac{2\pi }{eB}{E}_{N}.$$

When a magneto-exciton forms due to an inter-LL transition with *N* → *N* + 1 (*p* = 1), the change in circulation Δ*κ* = *κ*_*N*+1_ − *κ*_*N*_ = 2*π*$\hbar$*ω*_c_/*eB* = *h*/*m*_c_ is “quantised”. In superfluids, the circulation quantum can be envisaged as a “quantum” of viscosity^44,45^. For a sheet density of *n* = 3.2 × 10^12^ cm^−2^, *h*/*m*_c_ ≈ 0.02 m^2^ s^−1^. Therefore, at the onset of dissipation when *v*_d_ = *v*_c_, the dimensionless ratio *R* = *v*_c_*W*/Δ*κ* = *eBW*/2*hk*_F_(≈6 at *B* = 1 T), acts rather like a critical Reynolds number in classical hydrodynamics.

In conclusion, we have measured the oscillatory magnetoresistance of large area, high mobility monolayer graphene Hall bars over a range of currents up to 1 mA. They reveal three distinct phenomena: Hall field-dependent MPR, behaviour analogous to Mach supersonics at intermediate temperatures (40 K), and a critical Landau velocity at low temperatures (5 K). All three quantum phenomena can be described by a semiclassical generic equation for intra- and inter-LL transitions.

## Supplementary information

Supplementary Information

## Data Availability

Data that support the plots within this paper and other findings of this study are available from the corresponding authors upon reasonable request. Source data are provided with this paper.

## Source data

Source Data
