# Supplementary material for: Graphene’s non-equilibrium fermions reveal Doppler-shifted magnetophonon resonances accompanied by Mach supersonic and Landau velocity effects
Source: Nat Commun. 2021 Nov 4;12:6392. doi: 10.1038/s41467-021-26663-4 (PMC8568928; doi:10.1038/s41467-021-26663-4)
Supplement: Supplementary file 1 — Supplementary Information [file 41467_2021_26663_MOESM1_ESM.pdf]

Supplementary information: Graphene's non-equilibrium fermions reveal Doppler-shifted magnetophonon resonances accompanied by Mach supersonic and Landau velocity effects.

M.T. Greenaway,[1, 2, *] P. Kumaravadivel,[3, 4] J. Wengraf,[3, 5] L.A. Ponomarenko,[3, 5] A.I. Berdyugin,[3] J. Li,[6] J.H. Edgar,[6] R. Krishna Kumar,[3] A.K. Geim,[3, 4] and L. Eaves[2, 3, †]

[1]*Department of Physics, Loughborough University, LE11 3TU, UK*
[2]*School of Physics and Astronomy, University of Nottingham, NG7 2RD, UK*
[3]*School of Physics and Astronomy, University of Manchester, Manchester, M13 9PL, UK*
[4]*National Graphene Institute, University of Manchester, Manchester, M13 9PL, UK*
[5]*Department of Physics, University of Lancaster, Lancaster, LA1 4YW, UK*
[6]*Tim Taylor Department of Chemical Engineering, Kansas State University, Manhattan, KS, 66506, USA*

(Dated: October 15, 2021)

1. SUPPLEMENTARY NOTE 1: BROADENING OF PEAK IN r_y DUE TO *INTRA*-LL SCATTERING

The width of the broad peak in r_y, marked by green dots in Fig. 2a of the main text, and its amplitude both increase steadily with increasing B. This is consistent with the energy broadening of LL width, $\Gamma \sim \sqrt{B}$, observed in previous experiments and theoretical work [Ref. 40 of the main text]. Intra-LL scattering gives rise to an increase in the dissipative voltage, V_y with a maximum at $v_d = v_s$. Assuming the peak in V_y has a Gaussian form, as it broadens the peak in the differential resistance, $r_y = dV_y/dI$, will be shifted by a small amount

$$v_d = v_s \pm \frac{\Gamma(B)}{2k_F\hbar} \tag{1}$$

for negative and positive values of the current, I, The green dots in Fig. 2a of the main text show that Eq. 1 provides a good fit to the position of the broad peak in r_y for $B \approx 0.5$ T and $B \approx 2$ T when $\Gamma = \gamma\sqrt{B}$ with $\gamma = 0.5$ meV T$^{-1/2}$, which is in good agreement with the LL broadening parameters extracted from the full quantum simulations of MPR, as discussed in Ref. [31] of the main text.

2. SUPPLEMENTARY NOTE 2: CARRIER DENSITY DEPENDENCE OF MAGNETO-OSCILLATIONS AT LOW TEMPERATURES

Supplementary Fig. 1 shows the carrier density dependence of $r_y(B)$ at low temperature, $T = 5$ K. At low magnetic field, around ± 0.1 T, we observe a series of sharp peaks corresponding to the magnetic focusing (MF) of ballistic electron trajectories as discussed in the main text. In addition, each curve reveals a strong and broad maximum in the differential magnetoresistance at $B \sim 0.4$ T. This value corresponds closely with the elastic inter-LL transition resonance condition of $B = 0.41$ T given by Eq. (10) of the main text, see dotted vertical lines in Fig. 1. We suggest that the small differences in the magnetic field position of these peaks is due to the competing interaction of the elastic transitions and resonant magnetophonon emission, see discussion in main text. We find that the strength of the peak in $r_y(B)$ at $B = 0.41$ T decreases with increasing carrier density, due the increased separation of the LLs at low carrier densities. In our manuscript we focus on detailed measurements with a carrier density of $n = 3.2 \times 10^{12}$ cm^{-2} as we find this is the optimum n-value to reveal the interaction of the elastic process with the splitting and shifts of the MPR peaks, which become more defined at high carrier density, see Ref. [30] of the main text.

* m.t.greenaway@lboro.ac.uk
† laurence.eaves@nottingham.ac.uk

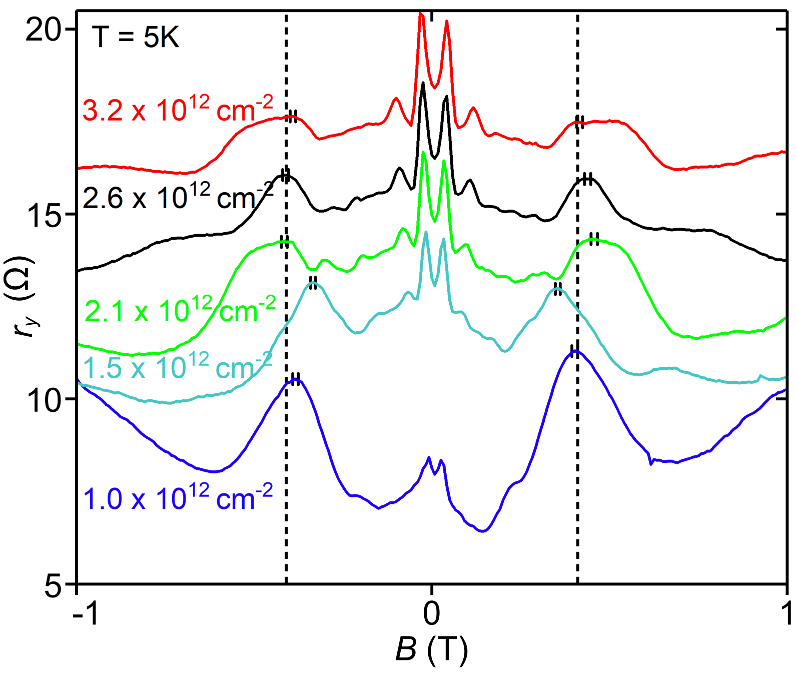

FIG. 1. Supplementary Figure 1 **Carrier density dependence of magnetoresistance oscillations** when $T = 5$ K and $I = -250$ µA for hole carrier densities ranging from $n = 1 \times 10^{12}$ cm^{-2} to $n = 3.2 \times 10^{12}$ cm^{-2}, labelled in the figure. Vertical dotted lines highlight the magnetic field-independent position of the resonance condition for elastic inter-LL transitions given by Eq. (10) of the main text. The magnetic field corresponding to the maximum in r_y is indicated by the small "H" marker. The oscillations around $B = 0$ arise from resonant magnetic focusing.
